# Surgical repair of rectovaginal fistulas: predictors of fistula closure

**DOI:** 10.1007/s00192-019-04082-w

**Published:** 2019-08-29

**Authors:** Jihong Fu, Zhonglin Liang, Yilian Zhu, Long Cui, Wei Chen

**Affiliations:** Department of Colorectal Surgery, Xinhua Hospital Affiliated to Shanghai Jiao Tong University School of Medicine, 1665 Kongjiang Road, Shanghai, 200092 China

**Keywords:** Rectovaginal fistula, Diverting stoma

## Abstract

**Introduction and hypothesis:**

We report the clinical outcome of surgical repair for rectovaginal fistula (RVF) carried out by one operative team. We also investigate the predictive factors for fistula healing.

**Methods:**

A retrospective cohort of 63 patients underwent local surgical repair of RVF during January 2008 and December 2017 by one operative group. The clinical features of the patients were reviewed. The association between fistula closure and diverse clinical parameters, including operative method, fistula location, prior repair, and diverting stoma, was analyzed.

**Results:**

Sixty-three consecutive patients underwent 80 local surgical repairs by our surgical team. Forty-five patients eventually healed after an average of 1.22 procedures. The overall success rate per procedure was 71.2%, whereas the closure rate of the first operation was 55.5% (*n* = 35). The etiology of the fistula did not impact on the success rate of surgical repair. The history of prior repair predicted a lower success rate on both overall procedure (RR = 0.59, 95% CI 0.41–0.85, *p* = 0.008) and the first repair in our institution (RR = 0.50, 95% CI 0.31–0.80, *p* = 0.003). There was no difference in closure rate between the stoma group and the non-stoma group. Nevertheless, among the 15 patients who underwent more than one operation in our center, a diverting stoma seemed to be necessary (10 patients healed in the stoma group and none of the patients healed in the non-stoma group, *p* = 0.02).

**Conclusions:**

History of prior surgical repair is a risk factor for failure. Diverting stoma did not increase the overall closure rate, but it seemed to be necessary for patients in whom the first operation failed.

## Introduction

Rectovaginal fistulas (RVFs) are abnormal epithelial connections between the rectum and vagina [1], leading to the passage of rectum content into vagina, which causes both physiological and psychological suffering to female patients. So far, RVFs remain a challenging pathological condition owing to the high failure rate of surgical repair. RVFs are usually caused by congenital malformation, obstetric injury, trauma, perianal sepsis, Crohn’s disease or are iatrogenic. Spontaneous healing of a fistula is rare and surgical repair is the main treatment for RVF.

Surgical repair of RVF is difficult and frustrating, although a series of surgical options are available for RVF, including advanced flap, muscle interposition, plugs, fistula excision. However, the success rate of surgical repair varies from 0 to 80% [2–6]. A large portion of patients undergo more than one surgical repair. Patients and surgeons are both plagued by the unsatisfying success rate as well as permanent stoma, impaired sphincter function, and recurrence after healing of the fistula.

The clinical outcome of RVF surgery could be affected by diverse factors, such as etiology, history of prior repair, the medical history of the patient, repair procedure, and diverting stoma. It has been reported that the failure of RVF repair is associated with Crohn’s disease and a history of smoking [5]. Several studies have claimed that a diverting stoma did not increase the healing rate of repair [5, 7], whereas one study found a diverting stoma necessary in radiation-related RVF [8]. However, fecal diversion was widely applied in RVF patients in clinical practice.

In this study, we aim to summarize the clinical outcome of RVF repair performed by our operative group and endeavor to identify the predictive factors of clinical outcome, ultimately providing more clinical evidence for treating RVF.

## Materials and methods

### Patient demographics

This study is a retrospective chart review analysis of patients who underwent surgical repair of RVF by one operative group (L Cui, W Chen, and Jh Fu) between January 2009 and December 2017. The exclusion criteria, including patients with high fistulas needing a trans-abdominal operation, patients who were treated only with a diverting stoma, and patients who were treated with seton.

The success of the repair was diagnosed at least 3 months after the last surgical repair or 3 months after closure of the stoma. Healing of the fistula was defined by the absence of any rectum content discharged from the vagina. Digital rectal examination or colonoscopy were further performed to confirm that the fistula was closed. Unclosed or recurrent fistula was defined as persistence of symptoms and was further confirmed by physical examination and colonoscopy. For patients in whom an un-closed fistula or recurrent fistula was suspected, a piece of gauze was placed in the vagina and a retention enema with methylene blue was carried out. The gauze was then removed 1 h after the enema and patients with blue gauze were diagnosed as having RVF.

Clinical data were retrospectively investigated including the following: patient demographics, history of prior repair, characteristics of the fistula, and the surgical repair procedure. To access the long-term outcome, a telephone interview was conducted on whether the patient still had symptoms related to RVF. The study was approved by the IRB of Shanghai Xinhua Hospital.

### Operative techniques

A trans-anal approach fistulectomy was performed as follows. For patients without a diverting stoma, bowel was prepared by oral intake of polyethylene glycol (PEGS). For patients with a diverting stoma, patients received a preoperative cleaning enema. Intravenous antibiotic prophylaxis was given 30 min before surgery (ciprofloxacin and metronidazole). The patient was placed in a jack-knife position and the assistants exposed the rectum using anal retractors. The fistula was identified by methylene blue or a probe. The fistula and scar tissue surrounding it were excised to guarantee that the margin was healthy tissue. Then a dissection in the recto-vagina septum was performed, and the rectal wall and the vaginal wall were dissected. The rectum wound was closed by absorbable suture longitudinally; the vagina wound was also closed or left open for drainage. Intravenous antibiotics were used for 72 h. For patients without a diverting stoma, liquid diet was used for 5 days to guarantee soft and deformed stools.

The advanced flap [8–10], Martius flap [11, 12], trans-perineal repair [13], and gracilis interposition [14] were performed, as previous described.

The decision regarding an ostomy for a diverting stoma was made by the senior author (L Cui) depending on the characteristics of the fistula, the history of previous repair, and the patient’s wishes. For patients requiring a diverting stoma, a laparoscopic-assisted sigmoid colostomy was performed.

For patients in whom the first repair from our institution failed, the next operation was performed at least 6 months after the first operation.

### Statistical analysis

The categorical data were analyzed using Chi-squared test or Fisher’s exact test. Student’s *t* test was used to compare continuous variables. *p* < 0.05 was considered statistically significant. All statistical analyses were performed using the statistical software program R 3.1.4. Odds ratio and risk ratio were calculated using the fmsb package.

## Results

### Patient demographics

Our study enrolled 63 patients who underwent 80 local repair procedures. The patients’ average age was 32 (range 16–67) years old, with a mean body mass index (BMI) of 21.4 (range 16.5–33.6). Thirty-four patients had undergone prior repair of RVF in other hospitals before admission to our department. The patient’s demographics are listed in Table 1.

**Table 1:** Demographics of the patients

Characteristic	*n* (%)
Patients	
Patient’s age	32 (16–67)
Prior repair	34 (53.9%)
Body mass index	21.4 (16.5–33.6)
Etiology	
Congenital	17 (26.98%)
- Obstetric injury	18 (28.57%)
- Iatrogenic cause	18 (28.57%)
- Sepsis	8 (12.69%)
- Trauma	2 (3.17%)
Trans-vaginal delivery	37 (58.73%)
Diabetes	2 (3.17%)
Smoking	2 (3.17%)
Alcohol usage	1 (1.59%)

Obstetric injury (*n* = 18, 28.57%) and iatrogenic causes (*n* = 18) were the most common etiologies of RVF, followed by congenital RVF (*n* = 17, 26.98%), sepsis (*n* = 8, 12.69%), and trauma (*n* = 2, 3.17%). The median duration of fistulas was 24 (range: 3–384) months. The characteristics of fistulas of each etiology are listed in Table 2. Iatrogenic RVFs had a higher frequency of higher location (above 2 cm from the dentate line), whereas more than half of the congenital fistulas are large in size (>1.0 cm).

**Table 2:** Etiological characteristics of fistulas

	Congenital	Obstetric injury	Iatrogenic	Sepsis	Trauma	Total
Location of fistula						
- ≤2 cm	12	13	8	6	2	41
- >2 cm	5	5	10	2	0	22
Diameter of fistula						
- <1.0 cm	6	11	11	7	1	36
- 1.0–2.0 cm	6	3	5	1	1	16
- >2.0 cm	5	4	2	0	0	11
History of previous repair						
- No	5	9	8	6	1	29
- Yes	12	9	10	2	1	34

Among the 18 patients with iatrogenically caused RVF, 13 patients had complications after low anterior resection (LAR) of the rectum, 2 after procedure for prolapse and hemorrhoids (PPH), whereas 3 fistulas were secondary to gynecological surgery. Three patients with congenital RVF had accompanying imperforate anus had undergone surgery in childhood. The other 15 patients had congenital fistulas without accompanying malformation.

The surgical procedures included trans-anal fistulectomy and suture of the rectum and vagina(*n* = 35, 43.75%), trans-anal advanced flap (*n* = 28, 35%), Martius flap (*n* = 10, 12.5%), gracilis interposition (*n* = 3, 3.75%), trans-perineal approach (*n* = 3, 3.75%), and trans-vaginal approach (*n* = 1, 1.25%).

### Clinical outcome

The median follow-up for the whole cohort was 50 (range 19–97) months. 45 patients were eventually cured after an average of 1.22 procedures. The overall success rate was 71.4%. Thirty-five patients were cured after the first repair and the success rate of first surgery was 55.5% (35 out of 63). The overall success rate and first repair success rate of diverse repair procedures were listed in Table 3. Further analysis suggested that tissue interposition (Martius flap or gracilis interposition) did not improve success rate compared with other procedures (7 out of 13 vs 38 out of 67,*p* = 0.9).

**Table 3:** Association between type of procedure and success rate

	Overall		First repair	
	Success	Fail	Success	Fail
Trans-vaginal approach	1	0	1	0
Trans-anal fistulectomy	20	15	13	13
Advanced flap	16	12	14	9
Martius flap	6	4	6	3
Gracilis interposition	1	2	0	2
Trans-perineal approach	1	2	1	1

Post-operative complications were observed in 17 procedures (21.25%), including 3 patients with urinary tract infection, 1 patient with pneumonia, 6 with wound dehiscence, 1 with rectal bleeding, and 6 with surgical site infection. One patient with rectal bleeding and 2 patients with surgical site infection required re-operation; the re-operation rate was 3.75%. No peri-operative mortality was observed.

To assess the risk of repair failure, multiple clinical factors were analyzed (Table 4). Clinical factors, including age, BMI, etiology, surgical procedure, smoking, and alcohol use, did not affect the closure rate of overall repair and the first repair. A history of previous repair indicated a lower success rate of fistula closure, both in overall procedure (RR = 0.59, 95% CI 0.41–0.85, *p* = 0.008) and the first repair (RR = 0.50, 95% CI 0.31–0.80, *p* = 0.003).

**Table 4:** Association between clinical characteristics and success of repair

	Overall procedure			First operation		
	Success	Fail	*p* value	Success	Fail	*p* value
Age	35.5 ± 12.3	35.1 ± 10.1	0.92	33.9 ± 11.5	35.9 ± 10.9	0.47
Smoke						
- Yes	2	2	1	1	1	1
- No	43	33		34	27	
History of trans-vaginal delivery						
- Yes	20	11	0.34	17	20	0.12
- No	25	24		18	8	
Body mass index	21.93 ± 3.5	21.57 ± 2.4	0.59	21.9 ± 3.6	21.4 ± 2.5	0.56
History of previous repair			0.01*			0.01*
- Yes	23	28		13	21	
- No	22	7		22	7	
Location of fistula			0.37			0.7
- High	15	16		11	11	
- Low	30	19		24	17	
Diameter of fistula			0.92			0.75
- <1.0 cm	26	21		21	15	
- 1.0–2.0 cm	12	8		9	7	
- >2.0 cm	7	6		5	6	
Etiology			0.70			0.30
- Congenital	12	10		10	7	
- Sepsis	6	3		6	2	
- Obstetrics	12	10		10	8	
- Trauma	2	0		2	0	
- Iatrogenic	13	12		7	11	

*p < 0.05

### The role of a diverting stoma in RVF surgery

A diverting stoma was usually required in patients with a RVF. In this study, we sought to identify the role of diverting stoma in RVF repair. In this cohort, 12 patients had a stoma before first admission and 15 patients underwent laparoscopic sigmoid colostomy; thus, 27 patients in total were operated on with a stoma, whereas 36 patients were operated without a stoma. Twenty-one patients ultimately healed with a stoma (21 out of 27, 77.8%) whereas 24 patients healed without a stoma (24 out of 36, 66.7%). No statistical significance was observed (*p* = 0.49).

All of the 21 patients who healed with a stoma underwent stoma closure operation within 3–12 months after repair. One patient manifested the symptom of recurrent fistula in 3 months after stoma reversal, and 3 patients developed wound infection, 2 patients had an ileus after the operation. No re-operation- or stoma reversal-related mortality was recorded.

We further sought to determine whether a diverting stoma is necessary in patients in whom a first attempt at repair failed. In this study, 15 patients in whom the first repair at our hospital failed underwent re-operation for local repair. For patients without a stoma, a diverting stoma was routinely recommended. Three patients declined colostomy and were operated on without a stoma whereas 12 did have a stoma. Ten of the 12 patients eventually healed, whereas none of the patients healed in the non-stoma group(*p* = 0.02). No statistical significance was observed between a successful operation and the size of the fistula (*p* = 0.77) or the location of the fistula (*p* = 0.61; Table 5). Thus, it was observed that a diverting stoma is probably necessary in patients in whom the first operation at our institution failed.

**Table 5:** Association between clinical characteristics and success rate in patients in whom first repair failed

	Success	Fail	*p* value
Location			
- High	4	3	0.61
- Low	6	2	
Stoma			0.03*
- Yes	10	2	
- No	0	3	
Size			0.38
- <1.0 cm	5	4	
- 1.0–2.0 cm	3	1	
- >2.0 cm	2	0	

*p < 0.05

## Discussion

In this study, we showed the characteristics and outcomes of local surgical repair of RVF patients with diverse etiologies. Three-quarters of the patients healed eventually whereas in one-quarter of the patients the repair failed. The overall success rate in our study in consistent with most of the previous reports on RVF local repair [5, 7].

In our study, the proportion of RVF etiology was quite different from that of previous reports [5, 7, 15, 16]. Congenital malformation, obstetric injury, and iatrogenic cause represented the majority (85%)of cases in our study, whereas inflammatory bowel disease is the leading cause of RVF in the literature [5, 7]. This inconsistency is probably because of the relatively low incidence of IBD in China.

Adult congenital RVF is common in China; these patients would not seek RVF repair until marriage age, probably because of the lack of prompt medical care and economic support. A large fistula usually manifested in adult congenital RVF patients, and an advanced flap or tissue transposition procedure is recommended to achieve tension-free reconstruction by healthy tissues.

Eighteen patients with iatrogenic RVF were included in our patient cohort, among which 13 were secondary to anastomotic leakage after low anterior rectal resection. The incidence of RVF after LAR was reported to be 3% by Watanabe et al. [17] LAR-related RVFs were treated individually. The reported management of these fistulas included conservative treatment, diverting stoma alone, local repair, and trans-abdominal operation. In our department, patients with a fistula in the lower part of the rectum were initially treated with local repair. A diverting stoma was usually constructed in the presence of anastomotic leakage before admission to our department. Regarding the situation, removal of the anastomotic staple pins and stitches surrounding the fistula was pivotal for healing.

Successful repair was determined by several factors. Features of the fistula, age, BMI, lifestyle, comorbidities, and history of previous repair directly affected the outcome. In our study, we demonstrated that the history of previous repair predicted a lower success rate in the first operation. Previous studies also implicated recurrent RVF as being the risk factor for failure. Lowry et al. reported that the success rate was 85% in the initial operation and this decreased to 55% at the third attempt [18] . A recent study carried out by Pinto et al. came to a similar conclusion that a history of prior surgery correlated with a higher failure rate [5]. Management of the recurrent RVF is even more difficult than initial repair. The scar tissue surrounding the fistula and the foreign body reaction of the stitches made the operation even more difficult. Some studies claimed that the interval between operations might enhance the success rate; however, in our study, for patients in whom the surgical repair failed, an interval of more than 6 months was routinely recommended to eliminate the local inflammation and edema. Smoking and diabetes were considered to influence the success rate of the operation according to common sense; however, owing to the smaller number of cases, such a correlation was not observed in our study.

A diverting stoma is a tough decision for both surgeons and patients. In our practice, patients with the following characteristics were usually advised to undergo a diverting stoma: fistula with a high location and large size; fistulas secondary to LAR; patients were supposed to undergo a gracilis graft; patients in whom the first attempt at our institution failed.

The role of diverting stoma in a successful surgical repair was controversial. Consistent with our result, the previous study did not reveal the relationship between the diverting stoma and the success rate [5]. However, most of authors of the published reports would still perform diverting stoma to treat RVF. Since the stoma did not benefit the overall success rate, it is not recommended as a routine procedure for RVF patients. Some studies suggest that a stoma should be considered in patients with a complex fistula or complicated recurrent fistulas [5, 19]. To answer the question who benefits from a diverting stoma, we further analyzed our data. For patients in whom the first operation in our department failed, although the sample size was limited, a diverting stoma seemed to be necessary to improve the success rate. Our patients who required a diverting stoma underwent a laparoscopically assisted sigmoid colostomy, which was minimally invasive and had higher acceptance by patients.

In this study, diverse surgical procedures for RVF repair were applied. The choice of procedure depended on the feature of the fistula, involvement of the sphincter, and the experience of the surgeon. It is hard to compose a standard algorithm on the procedure for selecting RVF patients, but there are some common concerns and principles when the decision is made.

Removal of the fistula tract, together with the surrounding scarred or granulomatous tissues, and the reconstruction of the septum by tension-free tissues with a good supply are the main principles of local surgical repair. A trans-anal approach, including an advanced flap, was frequently applied by the colorectal surgeon, whereas the trans-vaginal or trans-perineal approach was the prior choice of the gynecologist [19, 20]. According to our practice, advanced flap or tissue interposition were more favored for the first attempt in patients with a large fistula to achieve a good blood supply and tension-free reconstruction. Fistulectomy was widely used in our study. This operation was suggested for patients with a simple RVF or for those in whom the previous repair failed, but manifested a smaller fistula than before. The advantage of this operation was that it removed the septic fistula and the unhealthy tissue surrounding it, guaranteeing that the suture was applied to healthy tissues. The wall of the rectum, vagina, and rectovaginal septum was separated and reconstructed. A thick and enforced tissue was laid between the rectum and vagina. The reconstruction of the fistulectomy was similar to the trans-perineal approach; however, it avoided an incision on the perineum [12, 13]. With the development of trans-endoscopic microsurgery, such a procedure was reported to be performed with transmission electron microscopy, which provided better exposure and allowed for a precise operation [20, 21].

Repair of high RVFs is even more challenging and usually requires a trans-abdominal operation. In our institution, fistulas that could not be accessed via a local approach (trans-anal, trans-vaginal or trans-perineal) were recommended for trans-abdominal resection and low anastomosis. Owing to the distinct features of fistulas and surgical procedures, we did not include these patients in our study.

There were several limitations in this study. First, sphincter status was evaluated by clinical manifestation pre-operatively; imaging of sphincter defects was not routinely performed. Second, functional outcome was also not reported because the follow-up was carried by telephone interview. Since the operative group served in a colorectal surgery-specific department, the study was intented to include more complex fistulas and refractory fistulas. As mentioned above, the current study included a higher portion of adult congenital RVF patients and iatrogenic RVF patients. We also noticed that, compared with the western studies [5], we included more patients with RVFs larger than 1 cm. Despite the fact that congenital RVFs are usually large, this inconsistency may contribute to the selection bias of a tertiary referral center.

A prospective cohort study with a larger study size or a randomized clinical trial could be considered to testify our hypothesis that diverting stoma might promote the success rate of RVF repair.

In conclusion, in this study we report the clinical outcome of local surgical repair of RVFs in 63 patients. A history of prior surgical repair is a risk factor for failure of the first repair from our institution. A diverting stoma did not increase the overall closure rate, but it seemed to be necessary for patients in whom the first operation from our institution failed.
